# Chemical Composition and Antioxidant Properties of Peels of Five Pumpkin (*Cucurbita* sp.) Species

**DOI:** 10.3390/foods14122023

**Published:** 2025-06-07

**Authors:** Małgorzata Stryjecka

**Affiliations:** Department of Dietetics, The University College of Applied Sciences in Chełm, Pocztowa 54, 22-100 Chełm, Poland; mstryjecka@panschelm.edu.pl

**Keywords:** pumpkin peels, vitamins, amino acids, polyphenols, antioxidant properties, agro-food waste

## Abstract

By-products from the fruit and vegetable processing industry represent a substantial source of bioactive compounds, which can be extracted and utilized in the development of functional foods or nutraceuticals, thereby contributing to sustainable nutrition and waste valorization. Pumpkin peels are particularly abundant in bioactive components and contain significant fiber, protein, and minerals such as calcium and magnesium. This study determined the chemical composition, the content of water- and fat-soluble vitamins, and the antioxidant activity of peels from five pumpkin species: *Cucurbita pepo* ‘Kamo Kamo’, *C. maxima* ‘Bambino’, *C. moschata* ‘Butternut’, *C. argyrosperma* ‘Chinese Alphabet’, and *C. ficifolia* ‘Chilacayote Squash’. The highest moisture content was observed in the peels of *C. ficifolia* (89.2 mg 100 g⁻^1^ WW). In contrast, the highest amounts of protein (14.82 mg 100 g⁻^1^ DW), fat (1.59 mg 100 g⁻^1^ DW), and ash (7.46 mg 100 g⁻^1^ DW) were recorded in *C. maxima* peels. The peels of *C. moschata* contained the highest levels of total sugars (9.17 mg 100 g⁻^1^ DW), reducing sugars (8.48 mg 100 g⁻^1^ DW), and fiber (19.04 mg 100 g⁻^1^ DW). The peels of all analyzed pumpkin species were rich in amino acids and water- and fat-soluble vitamins. The highest levels of polyphenols and flavonoids and the most potent antioxidant properties (DPPH and FRAP) were found in the extract from *C. argyrosperma* peels. The findings of this study highlight the potential of pumpkin peels as a valuable source of bioactive compounds.

## 1. Introduction

In the 21st century, the fruit and vegetable industry generates large amounts of by-products with significant economic and practical potential. Almost 25–60% of the mass of vegetables and fruits consists of by-products. These waste materials serve as a source of bioactive chemical compounds that can be reused [1].

An example of such waste is pumpkin peels, which have an exciting chemical composition, making them a potential ingredient for food products [2]. Due to growing consumer concerns about synthetic additives in food production, the food industry is increasingly seeking plant-based materials that can be used to produce functional foods [3]. In Brazil, comparative studies on vegetable peels, pulp, and seeds have shown that peels and seeds contain higher amounts of nutrients, such as vitamins, minerals, and biologically active compounds, compared to pulp. For this reason, flours obtained from fruit and vegetable peels are used to produce baked goods (bread, biscuits, cereal bars). Adding fruit or vegetable peel flour to wheat flour improves the nutritional value of these products while reducing production costs.

Pumpkin peels contain significant amounts of fiber, protein, and minerals (calcium and magnesium) but significantly lower amounts of carbohydrates, lipids, and potassium than the pulp [4]. Additionally, pumpkin peels are a rich source of carotenoids, with high health potential [5,6,7]. Studies conducted by Nuerbiya et al. [8] have shown that pumpkin peels contain 10–40% of the total carotenoid content present in pumpkins. The variation in carotenoid levels in pumpkin peels found in the literature may be due to factors such as maturation stage, the climate conditions of cultivation, harvesting, and post-harvest processing.

Moreover, dietary fiber, specifically pectin from pumpkin peels, has been found to slow down starch digestion, which can be beneficial in managing diet-related diseases such as diabetes [9]. Additionally, literature reports indicate that pumpkin peel extracts slow down the oxidation of rapeseed oil [10]. Pumpkin peels have also been traditionally used in Persian medicine to treat ulcers, gastrointestinal bleeding, and wounds, including burns [11].

Furthermore, pumpkin peels contain high amounts of amino acids, including alanine, glycine, arginine, glutamic acid, aspartic acid, leucine, histidine, isoleucine, lysine, methionine, phenylalanine, threonine, serine, tyrosine, and valine [11]. Methanol and ethanol extracts from pumpkin peels exhibit antibacterial properties, with a 6–10 mm inhibition zone in the disc diffusion method. Jun et al. [12] extracted pectic polysaccharides from pumpkin peels and analyzed their effect on the growth of human gut bacteria. Their study concluded that pectic polysaccharides from pumpkin peels promote the growth of gut bacteria and lower glucose and bile acid levels, making pumpkin peels a promising functional food ingredient.

Therefore, this study aimed to determine the nutrient composition, amino acid content, minerals levels, vitamin content, and some antioxidant properties of the peels of five edible pumpkin species (*Cucurbita* sp.) grown in the temperate climate of southeastern Poland. Most of the existing literature focuses on the peels of the most common pumpkin species, *C. maxima* and *C. pepo*. In contrast, other *Cucurbita* species have not been thoroughly analyzed for their peel’s nutritional value. This study provides the first detailed description of the composition and properties of pumpkin peels from *C. argyrosperma* and *C. ficifolia.*

## 2. Materials and Methods

### 2.1. Plant Material

The study material consisted of the peels of five pumpkin species: *C. maxima* ‘Bambino’, *C. pepo* ‘Kamo Kamo’, *C. moschata* ‘Butternut’, *C. ficifolia* ‘Chilacayote Squash’, and *C. argyrosperma* ‘Chinese Alphabet’. The plants were cultivated in Chełm (latitude: 51°08′35″ N, longitude: 23°28′17″ E), Lublin Voivodeship, Poland, in 2022 under identical agro-climatic conditions.

Immediately after harvest, the pumpkins were washed and peeled. The peels were chopped into small pieces, frozen at −25 °C, and then lyophilized for 48 h using a FreeZone 12 L freeze dryer (Labconco Corporation, Kansas City, MO, USA) at a pressure of 0.37 mbar. The obtained freeze-dried pumpkin peels were stored at −70 °C until analysis.

### 2.2. Proximate Analysis

The content of basic components such as moisture, protein, ash, and fat in the studied freeze-dried pumpkin peels was determined in accordance with standard AOAC methods [13] (AOAC Volume II, 1990). The results were expressed as mg in 100 g DW.

Moisture content was determined according to AOAC Method 930.15, by drying the sample to a constant weight at 105 °C using an electric drying oven (ED 56, Binder, Tuttlingen, Germany). To determine the ash content, 3.0 g of lyophilized pumpkin peel from each species was weighed into a porcelain crucible and incinerated in a muffle furnace (LMH 07/12, Židlochovice, Czech Republic) at 550 °C (AOAC 942.05).

Total protein content was calculated based on nitrogen content, determined using the Kjeldahl method (AOAC 978.04), with a conversion factor of 6.25 (N × 6.25). The analysis used a Kjeltec™ 9 AutoSampler 60 (FOSS, Warszawa, Poland).

Crude fiber content was determined according to the AOAC gravimetric procedure (AOAC 920.860). Fat content in each dried sample was analyzed using a Soxhlet apparatus (Soxtec™ 8000, FOSS, Warszawa, Poland) with n-hexane as the extraction solvent (AOAC 2003.05).

All analyses were performed in triplicate.

### 2.3. Sugar Content

The total sugar content was determined using the phenol–sulfuric acid method [14], with slight modifications. For this purpose, 0.6 g of freeze-dried pumpkin peels from the studied cultivars were mixed with 0.6 mL of a 5% phenol solution and 1.0 mL of sulfuric acid. The resulting mixture was left to stand for 30 min, after which the absorbance was measured at 490 nm using a UV 2600i Plus spectrophotometer (Shimadzu, Tokyo, Japan). Distilled water was used as the blank, and glucose served as the calibration standard. For the determination of reducing sugars, the Nelson–Somogyi method was employed, with slight modifications [15].

### 2.4. Determination of Mineral Components

The sodium (Na) content was determined according to the methodology described by Abdualrahman et al. [16], using a flame photometer (Corning, Model 403, UK). In turn, for the determination of calcium (Ca), phosphorus (P), magnesium (Mg), potassium (K), iron (Fe), copper (Cu), and zinc (Zn), the method described by Ekpete et al. [17] was used, and measurements were conducted using an atomic absorption spectrophotometer (AA-7800, Shimadzu, Japan).

### 2.5. Determination of Amino Acid Content

The qualitative and quantitative composition of amino acids in the analyzed pumpkin peels was assessed using an amino acid analyzer (S433D, Sykam Co. Ltd., Eresing, Germany). One gramof lyophilized peels from the studied pumpkin species was subjected to hydrolysis using a 6.0 N HCl solution. This process was carried out in tightly sealed glass test tubes placed in an electric drying oven (SBS-ADO-2000, Steinberg^®^ Poland, Katowice, Poland) at a temperature of 110 °C for 24 h [18]. After this period, the samples were filtered through Whatman filter paper (Whatman 1444 150, Maidstone, UK) into a volumetric flask, which was then filled up to 100 mL with distilled water. The diluted samples were subsequently filtered through a 0.21 μm membrane to enable the quantitative determination of amino acids using the amino acid analyzer. The resulting amino acid values were expressed in g per 100 g of dry matter.

### 2.6. Vitamin Content

#### 2.6.1. Determination of B-Group Vitamins

B vitamins were determined according to the method described by Sami et al. [19], with slight modifications. To 2.0 g of freeze-dried pumpkin peel sample, 25 mL of 0.1 N H₂SO₄ solution was added, and the resulting mixture was heated for 30 min at 120 °C. The next step involved adjusting the pH of the mixture to 4.5 using a 2.5 M sodium acetate solution. Once the desired pH was reached, 0.05 g of diastase enzyme was added to initiate the enzymatic reaction, which was carried out for 12 h at 37 °C. After this period, the solution was diluted to a final volume of 50.0 mL with distilled water. The obtained solution was then filtered through a syringe filter (PES 33 mm, 0.45 µm, BioSens, Warsaw, Poland). Subsequently, 10 μL of the filtrate was injected into an HPLC system (Advanced Prominence-i, Shimadzu, Japan). Chromatographic separation was conducted using reversed-phase high-performance liquid chromatography (RP-HPLC). A C18 column (250 × 4.6 mm i.d., 5 μm) was used. The mobile phase consisted of solution A (phosphate buffer) and solution B (acetonitrile), applied in gradient mode. The gradient program for solution B was as follows: 0.0–3.0 min, B (1%); 3.1–10.0 min, B (50%); 10.1–16.0 min. The flow rate was set to 0.8 mL min⁻^1^, and the detection wavelength was 210 nm [20]. The described conditions were used for the determination of vitamins B1, B2, B3, B5, B6, and B9.

#### 2.6.2. Determination of Vitamin C

Quantitative determination of vitamin C was carried out according to the methodology described by Sami et al. (2014) [19], with minor modifications. To 5 g of freeze-dried pumpkin peel, 30.0 mL of a solution containing 0.3 M metaphosphoric acid and 1.4 M CH₃COOH was added. The resulting sample was placed in a 50 mL volumetric flask, mixed for 15 min (Fisherbrand™ Mini Vortex, Fisher Scientific, Waltham, MA, USA), and then filled up to a final volume of 50 mL. The solution was subsequently filtered using a filter (PES 33 mm, 0.45 µm, BioSens, Warsaw, Poland). The next step involved injecting 10 μL of the obtained filtrate into an RP-HPLC system. The standard solution of ascorbic acid was prepared using the same extraction solution. The separation was performed under isocratic elution conditions. The mobile phase consisted of solution A (0.1 M CH₃COOK, pH 4.9) and solution B (acetonitrile/water = 1:1), with a ratio of A to B being 33:67, a flow rate of 1.0 mL min⁻^1^, and a detector wavelength of 254 nm.

#### 2.6.3. Determination of Vitamins E, K and β-Carotene

To quantitatively determine vitamins E, K, and β-carotene, 10.0 g of lyophilized pumpkin peels was combined with 50 mL of 1 g pyrogallol, 70 mL of ethanol (C₂H₅OH), and 30 mL of 50% potassium hydroxide (KOH). The resulting mixture was transferred to a 50 mL volumetric flask and mixed using a Fisherbrand™ Mini Vortex (Fisher Scientific, USA) for 15 min. Subsequently, the solution was placed in a water bath at 50 ± 2 °C to complete the saponification process [20,21,22]. Extracts were obtained from three repetitions of the extraction process, each using a different volume of ethanol (20 mL, 30 mL, and 50 mL).

To neutralize the extract, demineralized water was used, while anhydrous sodium sulfate (Na₂SO₄) was employed to remove water from the extract. The extract was then concentrated to a volume of approximately 5–7 mL and diluted with methanol (CH₃OH) to a final volume of 10 mL. The next step involved injecting a 10 μL aliquot of the prepared sample into the HPLC system. The HPLC equipment was the same as that used for the analysis of K and E vitamins.

For the determination of β-carotene, a C8 column was used, with a mobile phase consisting of CH₃CN/CH₃OH/ethyl acetate (88:10:2), and detection was performed at a wavelength of 453 nm [23]. For the determination of vitamin E and K, a C18 column was employed with methanol (CH₃OH) as the mobile phase. The detection wavelengths for the various vitamins were as follows: α-tocopherol at 290 nm [24] and phylloquinone (K₁) at 244 nm (Sami et al., 2014) [19].

### 2.7. Total Phenolic Content

The extracts used for the determination of total phenolic content (TPC), total flavonoid content (TFC), and antioxidant properties were prepared by grinding 0.75 g of lyophilized pumpkin peels in a mortar with 25 mL of 80% methanol (Sigma-Aldrich, St. Louis, MO, USA), added in portions. The extraction time was 4 h. After this period, the samples were filtered through a funnel with a sintered glass disc into a 25 mL volumetric flask. The extraction process was conducted at 22 °C in the dark. For each pumpkin variety, three peel extracts were prepared. The extracts were stored in the dark at −25 °C.

The Folin–Ciocalteu method [25] was used to determine the total phenolic content in peel extracts of different pumpkin varieties. The procedure involved mixing 0.5 mL of the methanolic pumpkin peel extract with Folin–Ciocalteu reagent (10-fold diluted) and sodium carbonate (7.5%). These mixtures were then kept in the dark for 30 min. After this time, the absorbance of the resulting solution was measured using a UV-2600i Plus spectrophotometer (Shimadzu, Tokyo, Japan) at a wavelength of 760 nm. The total phenolic content was expressed in milligrams of gallic acid equivalents (GAE) per 100 g of dry weight (mg GAE 100 g⁻^1^ DW).

### 2.8. Total Flavonoid Content

To 0.5 mL of the methanolic extract obtained from the studied pumpkin peels, 2.0 mL of distilled water and 150 μL of a 5% NaNO₂ solution were added. The resulting solution was incubated in the dark for 5 min. After this period, 150 μL of a 10% AlCl₃ solution was added to the mixture, which was then incubated again in the dark for 6 min. Subsequently, 1.0 mL of a 1 M NaOH solution and 1.0 mL of distilled water were added to the reaction mixture, and the absorbance was measured using a UV-2600i Plus spectrophotometer (Shimadzu, Tokyo, Japan) at a wavelength of 510 nm [26].

The total flavonoid content was determined based on a quercetin calibration curve. The flavonoid content was expressed as quercetin equivalents (mg QE 100 g⁻^1^ DW).

### 2.9. Determination of Antioxidant Activity

The antioxidant activity was measured using the DPPH method, following the procedure described by Molyneux [27], with minor modifications. A volume of 50 μL of methanolic extract from pumpkin peels was mixed with 1.950 mL of a methanolic DPPH solution. The resulting mixture was thoroughly vortexed and then incubated in the dark at 25 °C for 30 min. After incubation, the absorbance of the samples was measured at a wavelength of 517 nm using a UV-2600i Plus spectrophotometer (Shimadzu, Japan). Trolox was used as the standard, and the results were expressed as µmol TE per 100 g dry weight (DW).

The antioxidant activity was also determined using the FRAP (Ferric Reducing Antioxidant Power) assay, based on the spectrophotometric method described by Benzie and Szeto [28], with slight modifications. The FRAP reagent was prepared by mixing 100 mL of acetate buffer (pH 3.6) with 10 mL of 10 mM TPTZ solution and 10 mL of 20 mM FeCl₃ solution. Then, 400 μL of the methanolic extract from pumpkin peel was mixed with 2.6 mL of the FRAP reagent. The resulting mixture was incubated at 37 °C for 30 min. After incubation, the absorbance was measured at 595 nm using a UV-2600i Plus spectrophotometer (Shimadzu, Japan). The results were expressed as µmol Fe(II) per 100 g dry weight (DW).

### 2.10. Statistical Analysis

All analyses were performed three times, and data were reported as mean with standard deviation (SD). All analyses were evaluated in Statistica version 12.0 (StatSoft, Krakow, Poland). The significance of means was tested post hoc with Tukey’s test combined with one-way analysis of variance (ANOVA); *p* < 0.05 was considered significant.

## 3. Results and Discussion

### 3.1. Chemical Composition

The chemical composition of the peels of five pumpkin species is presented in Table 1. The moisture content in the peels of the studied pumpkin species ranged from 88.5 mg 100 g⁻^1^ WW (*C. moschata*) to 89.2 mg 100 g⁻^1^ WW (*C. ficifolia*). The highest ash content was recorded for *C. maxima* (7.46 mg 100 g⁻^1^ DW), while the lowest was found in *C. ficifolia* (6.76 mg 100 g⁻^1^ DW). The peel of *C. maxima* also exhibited the highest protein (14.82 mg 100 g⁻^1^ DW) and fat content (1.59 mg 100 g⁻^1^ DW) among the examined samples. The fat content in the studied pumpkin peels was low (1.35–1.59 mg 100 g⁻^1^ DW), making them potentially suitable as food products for individuals who need to reduce fat intake in their diet, such as those with obesity, or as part of cardiovascular disease prevention [29,30].

The data on moisture, ash, protein, and fat content are consistent with those reported by Kim et al. (2012) [31] for Korean pumpkin (*C. maxima*) flour and with the findings presented by Amin et al. [32]. Meanwhile, fiber content ranged from 12.68 mg 100 g⁻^1^ DW (*C. pepo*) to 19.04 mg 100 g⁻^1^ DW (*C. moschata*).

The reducing sugar and total sugar content in the peels of the studied pumpkin species was significant, amounting to 7.48–9.17 mg 100 g⁻^1^ DW and 6.29–8.48 mg 100 g⁻^1^ DW, respectively. The highest total and reducing sugar content was found in *C. moschata*, while the lowest was in *C. ficifolia*. The results are also consistent with those presented by Amin et al. [32] for *C. maxma*.

The chemical composition of pumpkin peels is highly variable due to differences between species and/or cultivars of *Cucurbita* sp. cultivated in different regions worldwide.

### 3.2. Amino Acid Composition

The amino acid composition of the peels of five pumpkin species is presented in Table 2. In this study, 16 amino acids were identified. The peel of *C. maxima* had the highest content of the following amino acids among the analyzed species: alanine, histidine, leucine, glutamic acid, isoleucine, lysine, methionine, phenylalanine, serine, proline, threonine, valine, and tyrosine. Meanwhile, *C. pepo* contained the highest amount of arginine (1.15 g 100 g⁻^1^ DW), *C. moschata* had the highest glycine content (0.89 g 100 g⁻^1^ DW), and *C. ficifolia* had the highest aspartic acid content (3.01 g 100 g⁻^1^ DW).

The obtained amino acid contents are comparable to those reported by other authors [33,34]. Similar findings were obtained by Kim et al. [31] in their study on *C. maxima*, *C. pepo*, and *C. moschata* cultivars. However, significantly higher values were reported in the study by Jahan et al. [35].

### 3.3. Mineral Contents

The mineral content in pumpkin peels is influenced by many factors, including cultivar, species, climatic conditions during cultivation, and agricultural practices [36]. The highest concentrations of Mg, Na, K, Fe, and Zn were observed in the peels of *C. moschata*, while the highest levels of Ca, P, Mn, and Cu were found in *C. ficifolia* peels (Table 3).

All these minerals play a crucial role in human health. K, Ca, P, and Na are essential human growth and development macronutrients. Potassium is one of the most important electrolytes in the body, essential for cellular function. Potassium ions directly contribute to membrane potential formation, thus influencing the excitability of nerve and muscle cells [37,38].

The results suggest that pumpkin peels, particularly *C. moschata*, are rich in potassium and could be regularly consumed to help prevent or manage high blood pressure, reduce the risk of heart attacks and strokes, and lower mortality from heart diseases [39,40]. Calcium is essential for maintaining proper physiological functions in the human body, and diet remains the best source of calcium. According to the literature, the Ca content in pumpkin peel is 1.5 times higher than in seeds and four times higher than in the pulp [32]. Furthermore, research by Oyeyinka and Afolayan [41] showed that the Ca content in pumpkin peel was approximately 1.27 times higher than in banana peel. Significantly higher calcium contents than those presented in this paper were obtained in a study by Johan et al. [35], 6.87 mg 100 g^−1^, and Hussain et al. [1], 4.58 mg 100 g^−1^.

Phosphorus is one of the main components of nucleic acids and cell membranes, making dietary phosphorus intake essential for human health. Additionally, Ca and P are key components of human bone structure, and low levels of these minerals have been associated with osteoporosis. Therefore, regular consumption of pumpkin peels may effectively prevent osteoporotic fractures and reduce fracture risk in older adults [42]. Similar findings were reported by Amin et al. [32] in their studies on *C. maxima* peels.

### 3.4. Vitamin Content in the Peels of the Examined Pumpkin Species

Pumpkin peels serve as a reservoir of vitamins. They contain B-group vitamins (B1, B2, B3, B6, and B9) essential for energy metabolism and overall well-being. Green and yellow pumpkin peels contain significant amounts of vitamin C, which supports the immune system, and vitamin E, a powerful antioxidant. Vitamin K contributes to blood clotting and bone health.

Table 4 presents the vitamin content in the peels of the examined pumpkins. The order of vitamin content in the peels of the five studied pumpkin species, from highest to lowest, is as follows: V C > β-carotene > V B1 > V B2. The vitamin content in the tested peels is influenced by the cultivar, cultivation method, and climatic conditions. The vitamin C content in the examined pumpkin peels (Table 4) ranged from 8.475 mg 100 g^−1^ to 10.080 mg 100 g^−1^, with the highest concentration found in the peel of *Cucurbita maxima*. The levels reported in this study are slightly higher than those presented by Amin et al. [32], who analyzed different parts of pumpkins, including the peel of *C. maxima*. However, research conducted by Li et al. [43] on *C. pepo* showed a higher vitamin C content in the peels, reaching 15.1 mg 100 g^−1^. Vitamin C plays a crucial role in wound healing and scurvy prevention and may help prevent the conversion of nitrates into carcinogenic nitrites in the digestive tract [44].

The highest level of vitamin K was recorded in C. maxima peel, measuring 0.0017 mg 100 g^−1^. The peel of *C. pepo* had the highest content of vitamin B1 (0.089 mg 100 g^−1^). The highest vitamin A content was found in *C. moschata* peels (0.504 mg 100 g^−1^). The highest levels of vitamins B2 (0.227 mg/100 g), B9 (0.025 mg/100 g), and E (1.23 mg/100 g) were recorded in the peel of *C. ficifolia*. Meanwhile, the peels of *C. argyrosperma* contained the highest levels of vitamin B3 (0.81 mg 100 g^−1^), B5 (0.320 mg 100 g^−1^), and B6 (0.071 mg 100 g^−1^).

In many developing countries, agricultural production faces challenges due to inadequate farming systems, which may increase the risk of vitamin deficiency-related diseases [45].

### 3.5. Polyphenol Content and Antioxidant Properties of Pumpkin Peels

The total phenolic and flavonoid content in the peels of the analyzed pumpkin species is presented in Table 5. The content of these compounds in the tested peels ranged from 82.30 to 99.24 mg GAE 100 g^−1^ DW. The highest total phenolic content was observed in the methanolic extract of *C. moschata* peels, while the lowest was recorded in *C. ficifolia* extracts. The flavonoid content in the analyzed pumpkin peels ranged from 6.12 mg QE 100 g^−1^ DW (*C. pepo*) to 7.91 mg QE 100 g^−1^ DW (*C. argyrosperma*).

Jahan et al. [35], in their study on various parts of *C. maxima*, reported a polyphenol content of 9.13 mg GAE g^−1^ in the peel. The present study recorded twice the polyphenol and flavonoid content compared to pumpkin peels cultivated in Sri Lanka and Pakistan. Various factors influence the levels of phenols and flavonoids in plants, including cultivar, weather conditions, light intensity, ripeness, food processing, and preparation. Lower total flavonoid levels in methanolic extracts of *C. maxima* peels were reported by Dissanayake et al. [46].

Thus, the peels of different pumpkin species can serve as a rich source of nutrients, capable of meeting daily dietary needs. Their potential as dietary supplements to reduce oxidative stress is not only beneficial for health but also contributes to sustainable agricultural waste management. This dual role underscores the importance of our research in promoting responsible and sustainable practices in the field of nutrition and agriculture. The antioxidant properties, determined using the DPPH and FRAP assays, are presented in Table 5. In both tests, the highest activity was observed in *C. argyrosperma* peel extracts (24.62 µmol TE 100 g^−1^ DW and 76.74 µmol Fe (II) 100 g^−1^ DW for the DPPH and FRAP tests, respectively). The correlation between the results of both tests and the content of some chemical compositions confirms that their value significantly depends on the content of flavonoids and polyphenols (Table S1) Wanna [47], in a study including ethanol extracts from pumpkin peels, reported antioxidant activity levels of 37.04% (DPPH test) and 0.56 mM Fe^2^⁺g^−1^ extract (FRAP test). Meanwhile, Asif et al. [48] found that methanolic extracts from pumpkin peels exhibited an antioxidant potential of approximately 70%. Asif et al. [48], on the other hand, reported an antioxidant potential of approximately 70% for the methanolic extract of the examined pumpkin peels.

## 4. Conclusions

Nutritional composition analysis of pumpkin peels from five species revealed that all examined peels are rich sources of dietary fiber and essential minerals. They also exhibit high antioxidant activity, likely due to polyphenolic compounds, which was confirmed by calculating correlations.

The highest amounts of protein (14.82 mg 100 g⁻^1^ DW), fat (1.59 mg 100 g⁻^1^ DW), and ash (7.46 mg 100 g⁻^1^ DW) were recorded in *C. maxima* peels. The peels of *C. moschata* contained the highest levels of total sugars (9.17 mg 100 g⁻^1^ DW), reducing sugars (8.48 mg 100 g⁻^1^ DW), and fiber (19.04 mg 100 g⁻^1^ DW). The peels of all analyzed pumpkin species were rich in amino acids and water- and fat-soluble vitamins. The highest levels of polyphenols and flavonoids, as well as the most potent antioxidant properties (DPPH and FRAP), were found in the extract from *C. argyrosperma* peels. The findings of this study highlight the potential of pumpkin peels as a valuable source of bioactive compounds. Therefore, pumpkin peels can be used as a source of supplements or value-added products packed with nutrients and antioxidants. The results obtained in this study may be useful in evaluating the potential of the peels of the examined pumpkin species for applications in the cosmetic and pharmaceutical industries. This study emphasizes that plant components typically discarded in food production, such as pumpkin peels, possess high antioxidant potential and can be used to counteract oxidative stress-related damage, including aging processes.

Finally, the findings of this study can inspire farmers, food producers, and nutrition experts to properly utilize these agricultural by-products, contributing to the development of functional foods or nutraceuticals. The author of this paper hopes that the study presented will contribute to a more sustainable use of natural resources.

## Figures and Tables

**Table 1:** Proximate composition, total sugars, and reducing sugars (mg 100 g⁻^1^ DW only moisture WW) in the peels of five pumpkin species (*Cucurbita* sp.).

Factors	*C. maxima*	*C. pepo*	*C. moschata*	*C. ficifolia*	*C. argyrosperma*
Moisture	88.9 ± 0.015 b	88.7 ± 0.032 c	88.5 ± 0.025 d	89.2 ± 0.051 a	89.0 ± 0.093 b
Ash	7.46 ± 0.042 a	7.28 ± 0.039 b	7.13 ± 0.028 c	6.76 ± 0.024 e	6.93 ± 0.056 d
Fat	1.59 ± 0.006 a	1.44 ± 0.009 b	1.56 ± 0.030 a	1.48 ± 0.014 b	1.35 ± 0.025 c
Protein	14.82 ± 0.06 a	13.28 ± 0.06 c	14.26 ± 0.08 b	12.35 ± 0.05 e	12.79 ± 0.04 d
Fiber	15.16 ± 0.05 b	12.68 ± 0.04 e	19.04 ± 0.07 a	13.68 ± 0.1 d	14.48 ± 0.16 c
Total Sugar	8.21 ± 0.04 c	9.07 ± 0.04 a	9.17 ± 0.05 b	7.48 ± 0.06 e	7.82 ± 0.05 d
Reducing sugar	7.64 ± 0.09 c	8.13 ± 0.05 b	8.48 ± 0.04 a	6.29 ± 0.06 e	6.54 ± 0.04 d

The values in the rows marked with the same letter are not statistically significantly different at *p* < 0.05.

**Table 2:** Amino acid composition of the peels of five pumpkin species (*Cucurbita* sp.) (g 100 g⁻^1^ DW).

Amino acid	*C. maxima*	*C. pepo*	*C. moschata*	*C. ficifolia*	*C. argyrosperma*
Alanine	1.50 ± 0.03 a	0.74 ± 0.01 b	0.57 ± 0.03 c	0.78 ± 0.02 b	0.60 ± 0.02 c
Arginine	0.53 ± 0.06 b	1.15 ± 0.03 a	0.25 ± 0.02 c	0.25 ± 0.02 c	0.23 ± 0.01 c
Aspartic acid	2.41 ± 0.03 c	1.60 ± 0.03 d	2.84 ± 0.02 b	3.01 ± 0.04 a	2.83 ± 0.02 b
Glutamic acid	4.15 ± 0.05 a	2.00 ± 0.03 d	2.24 ± 0.02 c	2.36 ± 0.03 b	2.24 ± 0.02 c
Glycine	0.50 ± 0.01 c	0.25 ± 0.02 d	0.89 ± 0.01 a	0.80 ± 0.02 b	0.87 ± 0.02 a
Histidine	1.60 ± 0.03 a	0.78 ± 0.02 d	1.04 ± 0.02 c	1.13 ± 0.02 b	1.10 ± 0.02 bc
Isoleucine	1.67 ± 0.06 a	0.61 ± 0.02 b	0.55 ± 0.05 b	0.45 ± 0.02 c	0.57 ± 0.03 b
Leucine	1.74 ± 0.05 a	0.64 ± 0.01 c	0.88 ± 0.02 b	0.93 ± 0.02 b	0.88 ± 0.01 b
Lysine	0.98 ± 0.03 a	0.47 ± 0.01 d	0.73 ± 0.02 b	0.67 ± 0.02 c	0.72 ± 0.02 bc
Methionine	0.28 ± 0.03 a	0.26 ± 0.01 b	0.19 ± 0.01 c	0.16 ± 0.01 c	0.18 ± 0.02 c
Phenylalanine	0.96 ± 0.03 a	0.42 ± 0.01 c	0.65 ± 0.01 b	0.70 ± 0.02 b	0.67 ± 0.01 b
Proline	1.24 ± 0.02 a	0.38 ± 0.01 d	1.12 ± 0.03 bc	1.05 ± 0.01 c	1.17 ± 0.03 b
Serine	0.77 ± 0.03 a	0.45 ± 0.01 d	0.59 ± 0.02 bc	0.54 ± 0.02 c	0.63 ± 0.02 b
Threonine	0.65 ± 0.01 a	0.29 ± 0.02 d	0.35 ± 0.01 bc	0.30 ± 0.02 cd	0.37 ± 0.01 b
Tyrosine	0.47 ± 0.02 a	0.26 ± 0.01 c	0.29 ± 0.02 bc	0.24 ± 0.01 c	0.32 ± 0.02 b
Valine	1.31 ± 0.02 a	0.59 ± 0.01 c	0.73 ± 0.02 b	0.77 ± 0.02 b	0.54 ± 0.02 c
Total	20.76	10.89	13.91	14.14	13.92

The values in the rows marked with the same letter are not statistically significantly different at *p* < 0.05.

**Table 3:** Mineral content in the peels of selected species of pumpkin (mg 100 g⁻^1^ DW).

Minerals	*C. maxima*	*C. pepo*	*C. moschata*	*C. ficifolia*	*C. argyrosperma*
Ca	1.47 ± 0.03 bc	1.36 ± 0.03 d	1.50 ± 0.06 b	1.74 ± 0.04 a	1.38 ± 0.03 cd
Mg	3.47 ± 0.07 c	3.74 ± 0.07 b	4.01 ± 0.02 a	3.14 ± 0.05 d	3.26 ± 0.06 d
Na	9.46 ± 0.03 c	10.57 ± 0.36 ab	11.07 ± 0.06 a	10.33 ± 0.09 b	10.69 ± 0.18 ab
K	698.24 ± 1.63 c	749.03 ± 6.27 b	818.71 ± 5.28 a	715.17 ± 2.86 c	695.09 ± 4.31 c
P	1.52 ± 0.03 bc	1.42 ± 0.02 d	1.58 ± 0.03 b	1.74 ± 0.04 a	1.46 ± 0.035 cd
Fe	4.55 ± 0.1 e	5.66 ± 0.05 c	6.55 ± 0.12 a	6.22 ± 0.1 b	4.27 ± 0.04 d
Zn	0.17 ± 0.005 cd	0.16 ± 0.004 d	0.23 ± 0.017 b	0.27 ± 0.01 a	0.19 ± 0.005 c
Mn	0.35 ± 0.009 d	0.45 ± 0.01 c	0.50 ± 0.01 b	0.56 ± 0.03 a	0.52 ± 0.006 b
Cu	0.034 ± 0.003 bc	0.043 ± 0.004 a	0.026 ± 0.003 c	0.043 ± 0.004 a	0.036 ± 0.001 ab

The values in the rows marked with the same letter are not statistically significantly different at *p* < 0.05.

**Table 4:** Vitamin content (mg 100 g^−1^) in the peels of the analyzed pumpkin species.

Vitamin	*C. maxima*	*C. pepo*	*C. moschata*	*C. ficifolia*	*C. argyrosperma*
β-carotene	0.481 ± 0.007 b	0.413 ± 0.002 c	0.504 ± 0.006 a	0.375 ± 0.009 d	0.331 ± 0.006 e
B1	0.070 ± 0.005 b	0.089 ± 0.004 a	0.059 ± 0.003 b	0.040 ± 0.005 c	0.082 ± 0.004 a
B2	0.14 ± 0.013 c	0.18 ± 0.006 b	0.125 ± 0.005 c	0.227 ± 0.012 a	0.203 ± 0.012 a
B3	0.71 ± 0.015 c	0.72 ± 0.006 bc	0.65 ± 0.01 d	0.75 ± 0.015 b	0.81 ± 0.015 a
B5	0.282 ± 0.003 d	0.298 ± 0.001 c	0.249 ± 0.002 e	0.310 ± 0.002 b	0.320 ± 0.003 a
B6	0.055 ± 0.002 c	0.064 ± 0.002 b	0.051 ± 0.0015 c	0.067 ± 0.001 b	0.071 ± 0.0021 a
B9	0.019 ± 0.001 b	0.015 ± 0.002 c	0.022 ± 0.001 ab	0.025 ± 0.001 a	0.023 ± 0.002 ab
E	0.91 ± 0.04 cd	1.06 ± 0.05 b	0.97 ± 0.02 c	1.23 ± 0.04 a	0.85 ± 0.03 d
K	0.0017 ± 0.001 a	0.001 ± 0.0005 a	0.003 ± 0.001 a	n.d.	n.d.
C	10.080 ± 0.033 a	9.576 ± 0.028 c	9.316 ± 0.078 d	8.475 ± 0.043 e	9.862 ± 0.035 b

n.d.—not defined. The values in the rows marked with the same letter are not statistically significantly different at *p* < 0.05.

**Table 5:** Total flavonoid and phenolic contents, in addition to antioxidant properties (DPPH, FRAP), of peel extracts obtained from five pumpkin species.

Antioxidants	*C. maxima*	*C. pepo*	*C. moschata*	*C. ficifolia*	*C. argyrosperma*
Total phenolic (mg GAE 100 g^−1^ DW)	97.60 ± 0.09 c	88.75 ± 0.07 d	99.24 ± 0.07 a	82.30 ± 0.14 e	98.73 ± 0.11 b
Total flavonoids (mg QE 100 g^−1^ DW)	6.84 ± 0.09 c	6.12 ± 0.10 e	7.42 ± 0.07 b	6.54 ± 0.05 d	7.91 ± 0.05 a
FRAP (µmol Fe (II) 100 g^−1^ DW)	65.84 ± 0.13 c	54.52 ± 0.12 e	69.25 ± 0.10 b	62.48 ± 0.24 d	76.74 ± 0.70 a
DPPH (µmol TE 100 g^−1^ DW)	18.82 ± 0.12 c	14.72 ± 0.06 e	20.37 ± 0.06 b	17.52 ± 0.07 d	24.62 ± 0.04 a

The values in the rows marked with the same letter are not statistically significantly different at *p* < 0.05.

## Data Availability

The original contributions presented in the study are included in the article/Supplementary Materials. Further inquiries can be directed to the corresponding author.

## Supplementary Materials

The following supporting information can be downloaded at: https://www.mdpi.com/article/10.3390/foods14122023/s1, Table S1: Linear correlation between antioxidant activities and some chemical compositions.
