# Supplementary material for: Chemical Composition and Antioxidant Properties of Peels of Five Pumpkin (Cucurbita sp.) Species
Source: Foods. 2025 Jun 7;14(12):2023. doi: 10.3390/foods14122023 (PMC12191720; doi:10.3390/foods14122023)
Supplement: Supplementary file 1 [file foods-14-02023-s001.zip › foods-3585183-supplementary.pdf]

Table S1. Linear correlation between antioxidant activities and some chemical compositions.

	moisture	ash	fat	protein	fiber	total sugar	reducing sugar	polyphenols	flavonoids	FRAP	DPPH
moisture	1,000000	-0,602700	-0,296454	-0,605921	-0,549269	-0,968609	-0,942395	-0,504972	-0,018114	0,140576	0,150020
ash	-0,602700	1,000000	0,497076	0,847522	0,114725	0,590259	0,716436	0,453268	-0,239298	-0,277062	-0,297814
fat	-0,296454	0,497076	1,000000	0,761465	0,525254	0,279805	0,498202	0,136109	-0,233811	-0,206060	-0,321882
protein	-0,605921	0,847522	0,761465	1,000000	0,566493	0,536018	0,723412	0,648828	0,063290	0,035123	-0,035594
fiber	-0,549269	0,114725	0,525254	0,566493	1,000000	0,414596	0,490237	0,642654	0,553950	0,485418	0,405372
total sugar	-0,968609	0,590259	0,279805	0,536018	0,414596	1,000000	0,963309	0,340206	-0,166478	-0,334159	-0,331451
reducing sugar	-0,942395	0,716436	0,498202	0,723412	0,490237	0,963309	1,000000	0,405221	-0,175347	-0,319246	-0,341656
polyphenols	-0,504972	0,453268	0,136109	0,648828	0,642654	0,340206	0,405221	1,000000	0,739855	0,681197	0,669528
flavonoids	-0,018114	-0,239298	-0,233811	0,063290	0,553950	-0,166478	-0,175347	0,739855	1,000000	0,976031	0,977142
FRAP	0,140576	-0,277062	-0,206060	0,035123	0,485418	-0,334159	-0,319246	0,681197	0,976031	1,000000	0,990536
DPPH	0,150020	-0,297814	-0,321882	-0,035594	0,405372	-0,331451	-0,341656	0,669528	0,977142	0,990536	1,000000

Explanations: Statistically significant correlations are marked in red
